# St. Luke’s Hospital

**Published:** 1885-10

**Authors:** Frank Cary

**Affiliations:** 3027 Indiana Avenue, Chicago. Ill.


St. Luke's Hospital.—A Case of Multiple Abscess of the Liver. *Reported by* Frank Cary, M. D.

Matthew F., a medium-sized Irishman, of temperate habits, fifty-five years old, a stone-cutter by trade, was admitted to St. Luke's Free Hospital, September 1st, 1884, with a diagnosis of typhoid fever.

He gave a history of perfect health up to within three weeks of his admission to the hospital, when he began to suffer from headache, anorexia, profuse diarrhœa, and general malaise. There were no chills. The diagnosis of typhoid fever was questioned.

On admission, no tenderness could be detected in the region of the liver, or elsewhere over the abdomen. The patient, although very much exhausted, was not markedly emaciated. The stools were similar in appearance to those in typhoid fever of the third week. On the first day the temperature was normal. On the second day, by 6 P. M., it rose to 103°. On the third day it was normal in the morning, but gradually rose until 10 P. M., when the patient died, apparently from exhaustion.

Thn autopsy, twelve hours later, revealed the following conditions: The liver was completely riddled with abscesses, there being no less than twenty-four, varying in size from a hickory nut to a goose egg, and filled with dark, brownish pus, having no appreciable odor. The lobulus Spigelii was almost entirely destroyed by a large abscess. The organ, as a whole, was nearly normal in size; it was adherent to the diaphragm, the anterior abdominal wall, and to the transverse colon. There were different abscesses pointing in these several localities; of them, only one, that attached to the diaphragm and in the right lobe, seemed ready to rupture.

The colon, from the ileo-cæcal valve to the sigmoid flexure, was lined with ulcers. These coalesced so as nearly to obliterate the mucous membrane within these limits. The remaining intestinal tract was normal. The spleen was neither enlarged nor softened, as it was in a somewhat similar case reported by Dr. Peabody, of New York.* The gall bladder was normal, and contained the normal amount of bile. It should be stated that this patient had for years been a resident of Chicago, and had never lived in the tropics.

Are we not justified in believing that these intestinal ulcers and the abscesses of the liver stand in the relation of cause and effect? Coats, in his pathology (page 587), in discussing the point, says he thinks this view is not, however, beyond question.

3027 Indiana Avenue, Chicago. Ill.

---

* *Medical Record*, January 3d, 1885.

